# Metabolic Syndrome and Positive Frailty Screening: A Cross-Sectional Study with Community-Dwelling Older Adults

**DOI:** 10.14283/jarlife.2024.12

**Published:** 2024-05-27

**Authors:** M.C.B. de Souza, G. da Silva Rocha, E. de Souza Sampaio, P.C. de Oliveira Garcia Rodrigues, R.A. Vieira, A.F. Souza Gomes, T.R. Pereira de Brito

**Affiliations:** 1. Institute of Motricity Sciences, Federal University of Alfenas, Alfenas, Brazil; 2. Health and Sport Sciences Center, Federal University of Acre, Rio Branco, Brazil; 3. Faculty of Nutrition, Federal University of Alfenas, Alfenas, Brazil; 4. Faculty of Nutrition, Federal University of Alfenas, Alfenas, Brazil; 5. Nursing school, Federal University of Alfenas, Alfenas, Brazil; 6. Postgraduate Program in Health and Nutrition, Nutrition School, Federal University of Ouro Preto; Ouro Preto, MG, Brazil; 7. Faculty of Nutrition, Federal University of Alfenas, Alfenas, Brazil

**Keywords:** Cross-sectional studies, frail older people, frailty, metabolic syndrome, older adults

## Abstract

**Background:**

Metabolic Syndrome is a set of disorders that characterized by the association of three or more risk factors, like the obesity central, dyslipidemia, borderline blood pressure, hyperglycemia, and the increase of triglycerides. However, these factors also can be associated with pathophysiology of frailty.

**Objectives:**

verifying whether the metabolic syndrome is associated to the positive frailty screening in the older people.

**Design:**

Cross-sectional study. Participants: 443 older people living in Rio Branco, Brazil.

**Setting:**

Data collection was carried out in two stages: a personal interview and blood collection.

**Measurements:**

The diagnosis of metabolic syndrome was based on the criteria of the Third Report of the National Cholesterol Education Program Expert Panel on Detection, Evaluation and Treatment of High Blood Cholesterol in Adults. The frailty screening was performed using subjective questions validated in a previous study. Descriptive statistics and multinomial logistic regression were used for data analyses.

**Results:**

There was a predominance of female older people (69.07%), aged between 60 and 79 years (87.13%), with an income greater than or equal to one minimum wage (72.09%), no cognitive decline (75.94%) and depressive symptoms (63.31%), independent for BADL (86.46%) and dependent for IADL (51.69%). From the total sample, 56.88% of the older people were identified as frail, 34.09% pre-frail and 9.03% non frail. The prevalence of metabolic syndrome was 51.69%. After adjusting by the independent variables, an association between metabolic syndrome and pre-frailty was observed, and older people with metabolic syndrome were more likely to be prefrail (RRR=2.36; 95%CI=1.08-5.18).

**Conclusion:**

The metabolic syndrome was associated to the increase chance of screening for prefrailty in the older people evaluated, which reinforces the needy to establish preventive measures in relation to the metabolic syndrome to avoid frailty in the older people.

## Introduction

**A**ccording to the World Health Organization, the combination between common aspects of modernity, such as globalization, urbanization, and changes in the lifestyle, makes chronic non-communicable diseases one of the main causes of mortality (1). In this context, metabolic syndrome stands out, as it is a health condition strongly associated with behavioral issues, increasing the incidence of cardiovascular diseases and mortality (2).

Metabolic Syndrome (MetS) is a set of disorders that affect the cardiovascular system, characterized, according to the National Cholesterol Education Program’s Adult Treatment Panel III (NCEP-ATP III), by the association of three or more risk factors, like the obesity central, dyslipidemia, borderline blood pressure, hyperglycemia, and the increase of triglycerides (3, 4). Once the circulatory system is affected in the metabolic syndrome, some chronic diseases are commonly associated, such as atherosclerosis and coronary artery disease (5).

The prevalence of metabolic syndrome varies according to ethnic differences, sex and the criteria used to define the syndrome (4). For example, the World Health Organization (WHO) criteria consider the presence of type 2 Diabetes to be necessary, so when these criteria are used, the prevalence tends to be lower compared to the criteria of the International Diabetes Federation (IDF) or the NCEP-ATPIII (6).

However, the associated factors with MetsS are not limited only to cardiometabolic complications, and frailty may occur, for example, since characteristics of the metabolic syndrome, such as obesity and insulin resistance, may be present in the pathophysiology of frailty (7). Frailty is a geriatric syndrome with multiple causes, characterized by the decrease of strength, of the resistance and the physiological function, leading to increased vulnerability, functional loss, institutionalization, falls, and high risk for mortality (8, 9).

Due to the different definitions of frailty proposed, there are several ways to identify the syndrome, and the identification of phenotypes that require objective measures, such as grip strength, making this difficult to apply in clinical practice, especially in developing countries, where human resources may be scarce. Thus, alternative frailty assessment strategies have been elaborated to be applied in the clinical context, basing mainly on self-reported measures. These alternatives aim to capture the central aspects of frailty, maintaining predictive validity for adverse results, and may be useful for frailty screening (10, 11).

Despite the high prevalence of MetS and frailty, and the vast literature about these conditions, studies about the association between the two syndromes, especially among older people, are still low. Studies utilizing self-reported measures for frailty screening can be valuable for initiating early preventive actions targeting metabolic syndrome-associated frailty. Especially in developing countries, human resources for conducting assessments of older individuals, including physical measurements, may be scarce. Therefore, this study aims to investigate the association between frailty, measured through self-reported screening instruments, and metabolic syndrome in older individuals.

## Materials and Methods

### Study design and participants

This is a cross-sectional study, carried out in the municipality from Rio Branco, Acre, Brazil. Rio Branco is the capital of Acre, situated in the northern region of Brazil. It stands as the most populous and developed city in the state. We followed the Strengthening the Reporting of Observational Studies in Epidemiology (STROBE) reporting guidelines for cross-sectional studies (12).

**Figure 1.**
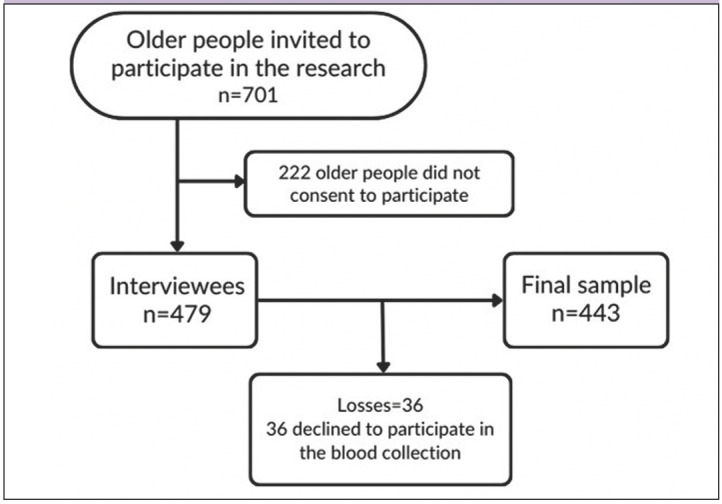
Sample definition

The calculation of the sample size was obtained considering the estimation of proportions in the order of 0.50, a confidence interval of 95%, a design effect (deff) of 1.17, and a population of 24.043 older people, resulting in a sample of 443 older people. The design effect is determined as the ratio between the variance of an estimate obtained through a specific sampling strategy and the variance of the identical estimate derived from a simple random sample comprising the same number of observational units (13). The deff of 1.17 was adopted based on previous study (14) (Figure 1).

The random selection of the sample was carried out from the records of the older people in the G-MUS System – Municipal Health Management, using Microsoft Office Excel. To perform the draw, the list of older people registered in November 2018 was used, which added up to 22.370 older people, that is, 89.3% of the population aged 60 years or older used to the sample size calculation.

Inclusion criteria were being aged 60 years or older, presenting neurological and/or cognitive conditions that enabled answering of the questionnaire (perceived by the interviewer during the presentation of the research and invitation to participate), and the absence of permanent or temporary inability to walk, except with the use of a walking aid device. Exclusion criteria were refusing to donate biological material (blood).

Data were collected from July to December 2019 at two different times. First, a home interview was carried out and, up to seven days after the interview, blood was collected at the older people’s home.

### Ethical aspects

All procedures were performed in accordance with the ethical standards of the Helsinki Declaration (as revised in Brazil 2013) and based on resolution 466/2012 of the National Research Ethics Committee in Brazil. This research was submitted to the Research Ethics Committee of Federal University of Acre, being approved in October 2017 under opinion No. 2.319.053. All participants signed an informed consent form and were able to clarify any possible doubts regarding their participation in this study.

### Study variables

#### Dependent variable

The dependent variable of this study was the self-reported frailty obtained through questions related to the components of this syndrome: unintentional weight loss, strength reduction, slowness (walking speed reduction), low physical activity and fatigue. The questions used were validated in a study carried out in Brazil (15). They were considered “frail” older people who scored for three or more components, “prefrail” those who scored positively for one or two, and “non frail” those who didn’t score in any of the components described.

#### Independent variable of interest

The independent variable of interest in the study was the metabolic syndrome identified as recommended by the NCEP ATPIII, which considers the combination of at least three components: abdominal obesity (measured by waist circumference, >102cm for men and >88cm for women), triglycerides ≥150mg/dL or the use of lipid-lowering drugs, HDL cholesterol <40mg/dL for men and <50mg/dL for women, blood pressure ≥130mmHg or ≥85mmHg or the use of antihypertensive drugs, fasting glucose ≥100mg/dL or previous diagnosis of Type 2 diabetes mellitus or the use of hypoglycemic agents (16).

#### Descriptive and adjustment variables

Descriptive and adjustment variables were sex (male; female); age group (60 – 79 years; 80 years and over); cognitive decline (no decline; with decline); depressive symptoms (no; yes); performance in Basic Activities of Daily Living (BADLs) (independent; dependent); and performance in Instrumental Activities of Daily Living (IADLs) (independent; dependent).

### Instruments used to collect information

To evaluate cognitive decline, depressive symptoms, and performance in BADLs and IADLs, the validated instruments described below were used.

Cognitive Abilities Screening Instrument – Short Form (CASI-S): an instrument designed to identify cognitive alterations in older people. The maximum score is 33 points and the cut-off point adopted for screening for cognitive decline is 23 (17, 18).

Geriatric Depression Scale (GDS): identifies the presence of depressive symptoms in older adults through 15 questions with yes/no answers. Positive screening for depressive symptoms is considered a score ≥ 6 (19, 20).

Katz scale: evaluates performance in BADLs. The BADLs consist of self-care tasks, including six functions: going to the bathroom, dressing, taking a shower, moving around, being continent (keeping control over eliminations), and eating (21). Older adults who performed all BADLs without assistance were considered independent.

Lawton & Brody scale: the scale evaluates the performance of the older adults in IADLs (22), which are adaptive tasks developed together with the community in an independent life and which include tasks such as using transport, doing household chores (taking care of the house and preparing meals), shopping, making phone calls, managing their own finances, and taking medication. Older people who performed all IADLs without assistance were considered independent.

### Data treatment and statistical analysis

The database was built in Microsoft Office Excel, version 2019 (16.0), with double data entry being performed in order to correct possible typing errors. Statistical analyses were performed using Stata software, version 13.0. In the descriptive analysis of the data, the proportions were estimated and the differences between the groups were identified using the Pearson’s χ2 test. For the association analysis, multinomial logistic regression was used. All independent variables were kept in the final model for adjustment. In all analyses, a significance index of 5% was used.

## Results

There was a predominance of female older people (69.07%), aged between 60 and 79 years (87.13%), with an income greater than or equal to one minimum wage (72.09%), with no cognitive decline (75.94%) and depressive symptoms (63.31%), independent for BADL (86.46%) and dependent for IADL (51.69%) (Table 1).

**Table 1.** Percentage distribution of the older people according to the socioeconomic, health and metabolic syndrome characteristics. Rio Branco, Acre, Brazil, 2019. (n=443)

Variable	Total n(%)	Non frail n(%)	Prefrail n(%)	Frail n(%)	p
Sex					
Female	306(69.07)	26(65.00)	107(70.86)	173(68.65)	0.757
Male	137(30.93)	14(35.00)	44(29.14)	79(31.35)	
Age Group					
60 to 79 years	386(87.13)	36(90.00)	131(86.85)	219(86.90)	0.850
80 years or more	57(12.87)	4(10.00)	20(13.25)	33(13.10)	
Cognitive Decline					
No	322(75.94)	35(89.74)	110(75.86)	177(73.75)	0.082
Yes	102(24.06)	4(10.26)	35(24.14)	63(26.25)	
Depressive Synptoms					
No	264(63.31)	31(86.11)	106(75.71)	127(52.70)	<0.001
Yes	153(36.69)	5(13.89)	34(24.29)	114(47.30)	
BADL^a^					
Independent	383(86.46)	38(95.00)	139(92.05)	206(81.75)	0.003
Dependent	60(13.54)	2(5.00)	12(7.95)	46(18.25)	
IADL^b^					
Independent	186(42.08)	26(65.00)	60(40.00)	100(39.68)	0.009
Dependent	256(57.92)	14(35.00)	90(60.00)	152(60.32)	
Metabolic Syndrome					
No	214(48.31)	26(65.00)	70(46.36)	118(46.83)	0.086
Yes	229(51.69)	14(35.00)	81(53.64)	134(53.17)	

a. BADL (Basic Activities of Daily Living); b. IADL (Instrumental Activities of Daily Living).

Positive frailty screening was identified in 56.88% of the older people, prefrail represented 34.09% and only 9.03% were classified as non-frail. The prevalence of metabolic syndrome was 51.69%.

Despite the proportion of older people with metabolic syndrome being higher among prefrail and frail, the test of difference in proportions wasn’t statistically significant. In the univariate multinomial regression analysis, it was observed that the older people with metabolic syndrome were more likely to be prefrail and frail (Table 2).

**Table 2.** Association between metabolic syndrome and frailty syndrome in the older people. Rio Branco, Acre, Brazil, 2019. (n=443)

	**Prefrail**			
	**RRRgross**	**CI95%**	**RRRadjusted**	**CI95%**
Male	0.76	0.36-1.59	0.89	0.39-2.01
80 years or more	1.03	0.98-1.09	1.02	0.96-1.08
Cognitive Decline	2.78	0.92-8.38	1.58	0.48-5.24
Depressive Symptoms	1.98	0.71-5.51	1.72	0.60-4.92
^a^ BADL Dependence	1.64	0.35-7.64	0.76	0.15-3.84
^b^ IADL Dependence	2.78	1.34-5.76	2.52	1.09-5.81
Metabolic Syndrome	2.14	1.04-4.43	2.36	1.08-5.18
	**Frail**			
	**RRRgross**	**IC95%**	**RRRadjusted**	**CI95%**
Male	0.84	0.42-1.71	1.16	0.53-2.55
80 years or more	1.01	0.96-1.06	1.00	0.94-1.06
Cognitive Decline	3.11	1.06-9.11	1.61	0.50-5.19
Depressive Symptoms	5.56	2.09-14.79	4.69	1.72-12.79
^a^ BADL Dependence	4.24	0.98-18.22	2.27	0.49-10.33
^b^ IADL Dependence	2.82	1.40-5.66	2.29	1.02-5.14
Metabolic Syndrome	2.10	1.05-4.22	1.81	0.84-3.89

a. BADL (Basic Activities of Daily Living); b. IADL (Instrumental Activities of Daily Living).

After adjusting by the independent variables, there was an association between metabolic syndrome and prefrailty, and the older people with metabolic syndrome were more likely to be prefrail (RRR=2.36; 95%CI=1.08-5.18). The dependence for IADL was associated with both prefrailty condition (RRR=2.52; 95%CI=1.09-5.81) and frailty (RRR=2.29; 95%CI=1.02-5.14). Depressive symptoms were associated only with the frailty condition (RRR=4.69; 95%CI=1.72-12.79) (Table 2).

## Discussion

This study aimed to investigate the association between frailty, measured through a self-reported screening tool, and metabolic syndrome in older people. The results indicated that metabolic syndrome was associated with an increased likelihood of pre-frailty in the evaluated older population, consistent with other studies that used objective measures to identify the frailty phenotype (7, 23, 24). Considering that the association between the two conditions was identified using a self-reported frailty screening tool, these findings are important in demonstrating that a simple and easily applicable tool can be used to screen frail older people in Brazil, which may help prevent metabolic syndrome.

A meta-analysis determined in the adult population in Brazil estimated, a prevalence of 42% when using the NCEP-ATPIII among the oldest (age ≥ 45 years) (25). As in Brazil, studies carried out in other countries with samples only of the older people are low. Similar results of prevalence were found in a study with 1099 Australians aged between 50 and 80 years, which estimated the prevalence of metabolic syndrome at 32% using the NCEP ATPIII criteria (26). On another hand, the prevalence of frailty in community-dwelling older people in Latin America and the Caribbean was 19.6% (95% CI: 15.4–24.3%) with a range of 7.7% to 42.6% in the studies reviewed, depending on the definition adopted (27).

A divergent result was found in a study that used data from the US National Health and Nutrition Examination Survey (NHANES) which found that younger people had a higher prevalence of metabolic syndrome and higher frailty index compared with the older people. This divergence of results can be explained by the fact that in the study with data from NHANES, different criteria were used to define MetS and frailty, and that the correlation analyzes were not adjusted for other variables (28).

Metabolic syndrome and frailty may share common pathophysiological mechanisms that put the older people at risk due to cardiovascular risk factors, coagulopathies, and metabolic deregulation (29). It is observed that the increase in blood pressure, a risk factor in MetS, can be correlated with a sedentary lifestyle, which in turn is associated with a decrease in functional capacity, and so can lead to a decrease in walking performance, making it slower. This decline in walking speed is present in the cycle of clinical manifestations of frailty (30).

Metabolic, immunological, and endocrine changes characteristic of MetS in older people may be related to the mechanisms of the frailty syndrome, since the chances of being prefrail or frail increased by about 50% with the presence of the MetS (7). The literature presents limited studies regarding the relationship between frailty and metabolic syndrome. The absence of similar research conducted with older people Brazilians complicates result comparisons. Nevertheless, it is noteworthy to mention a study conducted in Spain, revealing that. older people with MetS and compared with individuals without MetS presents an increase in the risk of frailty over a period from 3.5 years (23).

Regard to a possible explanation for the effect of metabolic syndrome about the frailty, didn’t find any association between the isolated components of MetS and frailty, suggesting that MetS as a set of disorders, rather than the sum of its parts, can increase the chances of frailty (31). The presence of hyperglycemia and hypercholesterolemia, as well as the use of drugs to treat these conditions, chronic inflammatory processes, and the catabolic state in an individual’s body, when associated, can cause clinical manifestations (31, 32).

These changes can lead to a loss of weight, muscle mass, energy, and walking speed, which are components of the Frailty Phenotype (9, 33). In addition, MetS is followed by peripheral insulin resistance, chronic microinflammation, activation of oxidative and prothrombotic mechanisms, and deregulation of the renin-angiotensin axis (31). All these mechanisms can have a detrimental effect on nutrition, as well as on the neuromuscular system and cognition of the older people. Furthermore, MetS has been associated to the higher occurrence and severity of microvascular brain damage, a condition that can accelerate cognitive and functional decline, leading individuals to the frailty (24).

The limitations of this study include the cross-sectional outline, which makes impossible the accomplishing causal inference and the difficulty in generalizing the results, since it is a sample of older people living in the Amazon region of Brazil. Furthermore, unfortunately, even with so much information available on the main physiopathological mechanisms of both syndromes, it was not possible to analyze, for example, blood biomarkers such as pro-inflammatory cytokines, immunological or hormonal profiles that could add more information to our study.

A strong point of this study is using a frailty tracking instrument that can be easily used by any professional in different health services. In addition to easy application and accessibility due to low cost, some evidence already indicates that the use of these tools can reliably contribute to frailty screening, which will make their use more common in the field of research and clinical practice (10, 11). Future studies may benefit from the use of these instruments and thus add to the body of existing evidence for their improvement. In the long term, this may enable the creation of guidelines that will add new elements to objective diagnostic methods.

## Conclusion

In conclusion, the metabolic syndrome was associated to the increase chance of screening for prefrailty in the older people evaluated. The results found from the use of this instrument in the present study indicate that frailty screening can be crucial among the older people with MetS, since frailty is a reversible process and that early interventions can prevent adverse outcomes potentiated by MetS. In addition, the diagnosis of MetS is easy and accessible, so that the identification of this condition in the older people can alert health professionals to the need for a more specific investigation of the health status of the older people, using instruments to identify frailty. Thus, the diagnosis of MetS in the older people can lead to a treatment that already considers the prevention of frailty.

## Data Availability

The data that support the findings of this study are available from the corresponding author upon reasonable request.
